# How Does Age at Diagnosis Influence Multiple Myeloma Survival? Empirical Evidence

**DOI:** 10.3390/healthcare13202637

**Published:** 2025-10-20

**Authors:** Michael O. Lawanson, Ernest Griffin, Daniel Berleant, Phillip Farmer, Ragen Hodge, Carolina Schinke, Cody Ashby, Michael A. Bauer

**Affiliations:** 1 Department of Information Science, University of Arkansas at Little Rock, Little Rock, AR 72204, USA; ewgriffin@ualr.edu (E.G.); jdberleant@ualr.edu (D.B.); 2 Department of Biomedical Informatics, University of Arkansas for Medical Science, Little Rock, AR 72205, USA; pfarmer@uams.edu; 3 Department of Biology, University of Arkansas at Little Rock, Little Rock, AR 72204, USA; rjhodge@ualr.edu; 4 Department of Internal Medicine, University of Arkansas for Medical Science, Little Rock, AR 72205, USA; cdschinke@uams.edu

**Keywords:** multiple myeloma, MM, age at diagnosis, survival outcome

## Abstract

**Background/Objectives:** Disparities in multiple myeloma (MM) survival occur based on factors like genetics, age, race, income level, and access to healthcare. The impact of age at diagnosis on MM survival is not fully understood and continues to draw research attention. This study explores the link between age at diagnosis and survival outcomes using data from the University of Arkansas Medical Sciences Myeloma Center Database (MMDB). **Methods:** Kaplan–Meier curves and Cox models were used to analyze the data. The log-transformed age variable strongly predicted survival. **Results:** The analysis found survival curves showing that patients in lower age brackets tend to have better survival profiles. Thus, for example, those in the oldest category (>70) showed the steepest decline, while the youngest age category (under 40) had better survival. Spline functions identified a non-linear relationship between age and survival. The likelihood ratio test, Wald test, and log-rank score test confirmed that the overall model was statistically significant, indicating that the spline-based approach effectively captured the relationship between age and survival. Further analysis using a stratified Cox model by age group showed significant risk differences. Patients aged 50–59, 60–69, and over 70 all had higher risks of death compared to younger patients, with those over 70 having a 3.3 times greater risk. **Conclusions:** In conclusion, the study confirmed that age at diagnosis has a significant association with survival outcomes for MM patients.

## 1. Introduction

Multiple myeloma (MM) is a plasma cell neoplasm associated with proliferation and buildup of dysfunctional plasma cells originating from the B-cell lineage [1,2]. In the U.S., MM ranks as the second most prevalent blood cancer, comprising roughly 1.8% of all cancer diagnoses overall and about 10% of hematologic malignancies [3]. Alternative estimates suggest it accounts for 1% of all cancers and 13% of blood-related cancers [4]. According to the American Cancer Society’s Cancer Statistics Center [5], the disease is most prevalent among older adults, with a median age at diagnosis of 69 years. The American Cancer Society [5] projects approximately 36,110 new MM diagnoses in the U.S. in 2025, with 20,030 cases in men and 16,080 in women. Additionally, approximately 12,030 deaths are anticipated, including 6540 men and 5490 women. The lifetime risk of developing MM in the U.S. is slightly less than 1%, or more specifically, roughly 1 in 108 men and 1 in 133 women.

MM typically progresses from precursor conditions, in particular monoclonal gammopathy of undetermined significance (MGUS), smoldering multiple myeloma, and plasmacytoma before advancing into the active stage of the disease [6]. According to current international guidelines, smoldering multiple myeloma should be managed with active monitoring, deferring treatment until the disease progresses to an active, symptomatic stage [7,8].

Once the disease is active, the stage of MM is also an important determinant of MM patient survival. The International Staging System, or ISS, can be used to help predict survival outcomes in MM patients. According to Palumbo et al. [9], the 5-year survival probability decreases with advancing stage: 82% for stage I, 62% for stage II, and 40% for stage III disease.

Despite substantial advances in treatment, MM remains a generally incurable disease [10], and survival outcomes vary significantly depending on multiple factors such as age, genetics, comorbidities, and access to care [11]. The prognosis of MM patients is influenced by various factors, including disease stage at diagnosis, cytogenetic abnormalities, overall health status, and notably, the age at which the diagnosis is made [12].

In recent decades, new treatment alternatives have both changed the clinical course of the disease and improved survival [2]. MM five-year relative survival rate has improved significantly over the past few decades due to advancements in novel therapeutic agents [12]. According to the National Cancer Institute’s Surveillance, Epidemiology, and End Results (SEER) Program, the five-year survival rate for MM patients in the U.S. has increased from 25% in the 1970s to approximately 56% as of 2023 [13].

There is increasing research attention on the implications of age at diagnosis on treatment and survival outcomes in MM. Previous population-based studies in Sweden, covering patients diagnosed up to 2003, indicated higher survival rates among those under 70 at diagnosis [14]. Research from the U.S. demonstrated better long-term survival for patients aged 65 to 80 at diagnosis, along with reduced early mortality across all age groups in more recent years [15]. A study analyzing 8239 newly diagnosed MM patients found that those who underwent ASCT (autologous stem cell transplantation) had a mean age of 58.1 years, compared to 67.9 years for those who did not receive ASCT.

Survival rates in MM have improved over recent decades across all age groups, though disparities persist. According to Luciano et al. [16] the five-year relative survival rate (RSR-5) increased from 38.2% to 61.8% for patients younger than 65 years (*p* = 0.001), from 29.0% to 48.4% for those aged 65 to 74 years (*p* = 0.001), and from 21.1% to 34.0% for patients 75 years or older (*p* = 0.001), between the periods 1993–1997 and 2008–2012; these results highlight the impact of age on treatment eligibility and choices [16].

Despite the improvements, older patients continue to experience lower survival rates compared to younger patients, highlighting the need for age-specific treatment strategies and supportive care interventions. For instance, younger patients (<60 years) have significantly higher survival rates compared to older adults. Younger patients are more likely to have better tolerance for aggressive treatment options, including high-dose chemotherapy and autologous stem cell transplantation (ASCT), and less comorbidity and death from other causes. However, the complete biological picture of the disease in younger compared to older patients is mixed [17]. For example, younger patients tend to have a higher rate of light chain disease, which tends to be more aggressive, yet also tend to be diagnosed earlier in the disease process [18]. Understanding the nuanced impact of age at diagnosis on survival may be crucial for future advances in tailoring treatment approaches and improving prognostic assessments in MM patients.

There is still insufficient evidence regarding the connection between age at diagnosis and survival outcomes. This motivates further research to clarify the effect of age at diagnosis on survival disparities between different age groups. Investigating the relationship between age at diagnosis and survival in MM patients is imperative, as age-related factors, including physiological resilience and comorbidities, can influence treatment tolerance and efficacy. Establishing this relationship remains a public health priority to improve MM survival rates. Also, incorporating age-specific survival data can enhance prognostic models, enabling clinicians to provide more accurate information to patients regarding their disease trajectory and expected outcomes.

The main purpose of this study is to evaluate the effect of age at diagnosis on the survival of patients with MM. This study will also evaluate alternative functional forms of age, including linear, log-transformed, and non-linear spline models to capture potential non-linear effects, addressing a common limitation of previous research that modeled age solely as a linear or categorical variable.

## 2. Methods

### 2.1. Data Source and Cohort

The dataset for the study was obtained from the University of Arkansas Medical Sciences (UAMS) long-standing Myeloma Center Database (MMDB) [19]. The cohort included 2743 patients diagnosed between 2005 and 2020, all of whom had available genomic profiling data. This analysis was intentionally focused on isolating and assessing the prognostic impact of age at diagnosis. Consequently, data on other potential prognostic factors such as specific treatment regimens (e.g., autologous stem cell transplantation (ASCT)), cytogenetic abnormalities, and performance status (e.g., ECOG score) were not incorporated into the current model. The relevance of these variables is acknowledged as an important direction for future research.

### 2.2. Variables and Outcome Measures

This analysis focused on three primary variables: age at diagnosis, overall survival (OS) time, and event status (death). OS time was defined as the duration from diagnosis to death, with all-cause mortality used as the endpoint. For individuals who were alive or lost to follow-up, survival data were censored at the date of their last recorded contact. Limiting the model to these variables ensures a clear and focused evaluation of age as a prognostic indicator, avoiding additional complexity beyond the scope of this specific aim.

### 2.3. Statistical Analysis

All statistical analyses were conducted in R (version 4.3.1) within the RStudio (current release as of August 2025) environment, employing the **survival, survminer, readxl, dplyr, officer,** and **flextable** libraries.

**Survival Analysis**: The Kaplan–Meier approach was employed to estimate overall survival rates. Patients were categorized into five distinct age groups for comparison: <40, 40–49, 50–59, 60–69, and ≥70 years. Survival curves were compared using the log-rank test, with statistical significance defined as a *p*-value less than 0.05.

### 2.4. Cox Proportional Hazards Modeling

To evaluate the functional relationship between age and survival, the ggcoxfunctional() function from the **survminer** package (ver. 0.5.0) was utilized. This function generates a plot of the continuous Age at Diagnosis variable against martingale residuals from a null Cox model. The LOESS (Locally Estimated Scatterplot Smoothing) curve from this plot was assessed to determine whether a linear, log-transformed, or spline term for age was most suitable for inclusion in the Cox model.

## 3. Results

**Figure 1:**
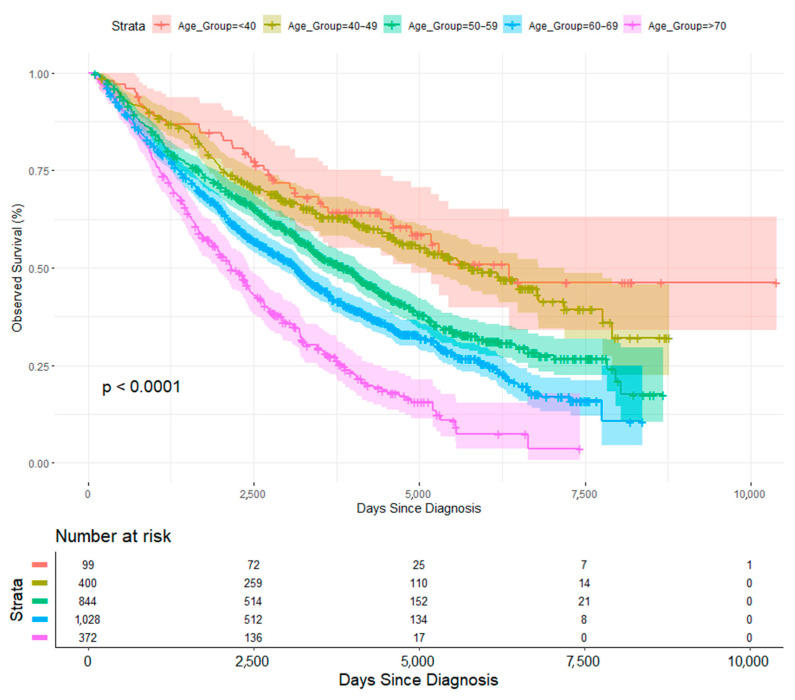
The Kaplan–Meier survival curves of multiple myeloma (MM) patients by age group.

The survival curves (Figure 1) demonstrate that patients in lower age brackets generally tend to have higher survival probabilities, while those in the older categories show steeper declines. The log-rank test (*p* < 0.0001) indicates a significant difference among the age groups. The risk table beneath the plot reveals how many participants remain at risk over time, with consistent attrition as follow-up extends. Younger patients maintain relatively better survival rates, whereas older patients exhibit more rapid declines. The gaps between the curves are not uniform, implying that the relationship between age and all-cause mortality risk may be non-linear. Additionally, the shapes of the curves differ across age groups, which points to the possibility of non-linearity in how age impacts outcomes. The most pronounced drop occurs in patients over 70, reflecting an age-dependent escalation in risk, possibly reflecting mortality from other causes.

Age at diagnosis plays a crucial role in multiple myeloma (MM) survival, and the KM curves suggest that a simple linear model may not fully capture the complexity of this effect. Therefore, applying log transformation or a Cox model with non-linear terms, such as restricted cubic splines or polynomial terms, may be more appropriate to describe the survival pattern.

### 3.1. Cox Proportional Hazard Models

**Figure 2:**
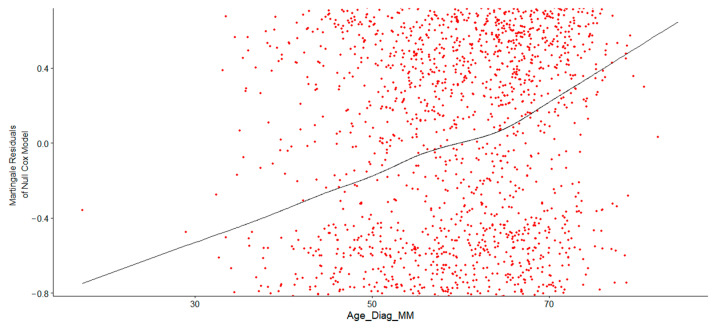
Martingale Residual Plot for Age at Diagnosis, showing that increasing age at diagnosis is associated with increasing log hazard. The red dots are individual residuals, while the black curve shows their overall trend using LOESS smoothing. The curve shows nonlinear curvature starting after age 50, indicating the increase becomes nonlinear.

Figure 2 shows the verified and correct specification of Age at Diagnosis in the Cox proportional hazards model. Consider the LOESS curve (black line). The curve in the trend line indicated a nonlinear relationship between Age at Diagnosis and the log hazard, suggesting that a simple linear term might not adequately capture this association. Consequently, a transformation, such as a logarithmic or polynomial function, or the use of restricted cubic splines (RCS), was necessary to properly fit the model. Initially, a log transformation was applied to Age at Diagnosis, and the Cox regression model output confirmed that log-transformed Age at Diagnosis was a significant predictor of survival time, with event status determining whether death occurred. The results demonstrated that log-transformed age was highly significant in predicting survival. The hazard ratio remained statistically significant, as its entire confidence interval was above 1, indicating that higher log-age consistently increased the hazard. Older patients (with higher log-age) exhibited significantly poorer survival outcomes.

**Figure 3:**
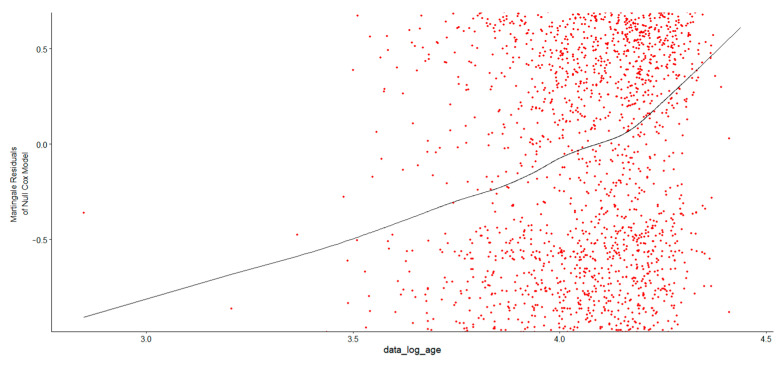
Martingale residual vs. covariate plot using Age at Diagnosis. The horizontal axis is not age as in the previous figure but the natural log of age, accentuating the nonlinearity which may be captured using the spline approach.

In Figure 3, after examining the Martingale residual plot to verify whether the log transformation was appropriate, the LOESS curve displayed an increasing trend, indicating that the residuals were not centered around zero. This suggested that the log transformation of Age at Diagnosis might not fully capture its relationship with survival time and that a nonlinear pattern exists, which was thus not well-represented by a simple log transformation of Age at Diagnosis. To address this, an alternative approach was considered using restricted cubic splines in R to model the nonlinear relationship between Age at Diagnosis and survival in MM patients. The log of Age at Diagnosis was modeled using natural splines with three degrees of freedom. The likelihood ratio test, Wald test, and log-rank score test confirmed that the overall model was statistically significant, indicating that the spline-based approach effectively captured the relationship between age and survival. The spline terms provide a nonlinear relationship between log of Age at Diagnosis and survival (Table 1).

All three spline terms were statistically significant, confirming a nonlinear relationship between Age at Diagnosis and survival. The high hazard ratios (HRs) indicated that survival decreased as Age at Diagnosis increased, but the effect did not follow a simple linear trend.

### 3.2. Proportional Hazards Assumption Test

After evaluating the proportional hazard (PH) assumption to determine whether the RCS (restricted cubic splines) model provided a better fit, the study observed a borderline violation (*p* = 0.049) for the spline-transformed log of (Age at Diagnosis). This suggested that the effect of Age at Diagnosis on hazard might have changed over time, potentially requiring model adjustments. Similarly, the global test yielded *p* = 0.049, indicating a borderline violation of the PH assumption for the overall model. Although this was not a severe violation, it still warranted further checks. Hence, we checked for an alternative model, in which we treated Age at Diagnosis as a continuous predictor. The study then categorized age at diagnosis into groups and stratified the Cox model to address the continual violation of PH assumptions in the models, because stratification removes the need to explicitly model the non-linear effect of age on survival.

As shown in Table 2, stratifying by age in a Cox model allows for separate baseline hazard functions across age groups, effectively controlling for non-linear effects by removing the need to assume a specific functional form for age. However, this approach does not yield a direct hazard ratio (HR) for age, as age is used solely to define strata rather than as a covariate in the model.

### 3.3. Stratified Cox Proportional Hazards Model and PH Test Results

The results from the stratified Cox proportional hazards model provided insights into the relationship between age group and survival in a cohort of 2743 patient observations, with 1619 observed events. By stratifying the model based on age groups, the study found statistically significant hazard ratios for the 50–59, 60–69, and 70+ age groups, as reflected in their *p*-values (*p* < 0.001). As represented in Table 3, the stratified Cox model results indicate that individuals aged 70+ faced a 3.3 times higher risk of experiencing the event compared to the reference group (patients under 40). Furthermore, the likelihood ratio, Wald, and score tests all confirmed a highly significant overall effect of age group on survival (*p* < 2 × 10^−16^). The concordance statistic of 0.577, with a standard error of 0.007, suggested a moderate ability of the model to distinguish between individuals with different survival times.

Given the model’s moderate discriminatory ability (C-index = 0.577), we explored whether the effect of age was influenced by another variable, gender. An analysis incorporating an interaction term between age group and gender revealed no statistically significant effect modification (*p* > 0.05), suggesting that the elevated hazard associated with older age was consistent across both genders.

## 4. Discussion

Age at diagnosis remains a significant predictor of survival in multiple myeloma (MM) patients, with younger individuals demonstrating superior outcomes. The present study found survival curves demonstrating that patients in lower age brackets (<40) tend to have higher survival probabilities, while those in the oldest category (70+) show the steepest decline. Generally speaking, existing research noted earlier shows that MM patients diagnosed before age 65 have significantly higher survival rates than those diagnosed later. This trend is reflected in other cancers as well, such as prostate cancer [20]. More generally, age at diagnosis remains one of the strongest predictors of survival time [21].

Kaplan–Meier survival curves have been widely used to illustrate differences in MM survival based on age groups. Fonseca et al. (2020) [22] analyzed data from the Surveillance, Epidemiology, and End Results (SEER) program and found that five-year overall survival rates for patients aged ≤60 years were significantly higher (62%) compared to those aged 70+ (34%). The log-rank test confirmed significant differences in survival distributions (*p* < 0.001). A study by Langseth et al. [23] found that older age was associated with lowered MM survival times.

This survival gap is influenced by a combination of treatment-related, biological, and host-specific factors. Firstly, older patients are less likely to tolerate intensive treatments or undergo autologous stem cell transplantation (ASCT). Consequently, younger MM patients are more likely to receive high-dose chemotherapy and ASCT, significantly improving survival. More generally, older patients often require dose reductions or alternative therapies, leading to suboptimal treatment responses [24].

Secondly, the biology of MM often varies with age; older individuals more frequently exhibit certain high-risk cytogenetic abnormalities such as del(17p) and t(4;14), which are associated with more aggressive disease and resistance to therapy [7]. On the other hand, genetic profiling of MM tumors in younger patients shows a higher prevalence of favorable cytogenetic markers like t(11;14).

Thirdly, host-related factors play a crucial role. Age-associated immune decline (immunosenescence) may impair the effectiveness of immunomodulatory therapies, while common comorbidities like renal dysfunction or cardiovascular disease can both limit treatment options, contributing to reduced treatment effectiveness and increased mortality risk [25], and independently contribute to mortality. Collectively, these factors help explain the observed disparity in survival between younger and older patients.

The result of the Cox model demonstrated that log-transformed age was highly significant in predicting survival. The hazard ratio remained statistically significant, as its entire confidence interval was above 1, indicating that higher log-age consistently increased the hazard. These older patients exhibited significantly worse survival outcomes. Several studies have applied Cox proportional hazards regression to assess the impact of age at diagnosis on MM survival. Other studies employing Cox proportional hazards models have consistently shown that age at diagnosis is a statistically significant factor influencing survival outcomes. For example, a model by Pettersson et al. [26] found that every additional year of age at diagnosis increased the hazard ratio for mortality by 1.08 in prostate cancer patients. For MM patients, a large-scale analysis by Kumar et al. [27] found that each additional year of age at diagnosis increased the hazard ratio (HR) for mortality by 1.06 (95% CI: 1.04–1.08, *p* < 0.001), confirming that older age is an independent predictor of shorter survival. Similarly, Anagnostopoulos et al. [28] confirmed that patients diagnosed with MM over the age of 70 had higher mortality compared to those diagnosed under age 70.

Empirical evidence and scholarly literature strongly support that younger age at cancer diagnosis is associated with higher survival probabilities. This advantage can likely be attributed to superior treatment tolerance, fewer comorbidities, early detection, and more effective physiological responses to therapy. Additionally, statistical models reinforce the significance of age as a predictor of survival, demonstrating its critical role in cancer prognosis.

These findings have significant clinical implications. The pronounced disparity in survival, most notably the 3.3-fold increased mortality risk among patients over 70, underscores the need for age-tailored treatment approaches. Age emerges as a strong prognostic indicator that must be factored into clinical decision-making. For older patients, particularly those with comorbidities, this may mean prioritizing treatment regimens that balance efficacy with tolerability. This could include dose-adjusted novel agents and enhanced supportive care, rather than therapies that may be too intensive or poorly tolerated. Conversely, in younger and fitter patients, the results reinforce the value of more aggressive, potentially curative strategies, such as autologous stem cell transplantation. From a prognostic standpoint, these data offer a meaningful framework for patient counseling, helping set realistic expectations and inform shared decision-making, whether the primary focus is life extension or quality of life. While age itself cannot be changed, a deeper understanding of its impact is a critical step toward more personalized and effective management strategies across the age continuum.

## 5. Conclusions and Future Work

Advances are continuing in the treatment of multiple myeloma (MM) patients, yet the disease remains largely incurable. While the inverse association between age and survival is known, its precise functional form is not well-characterized. Consequently, we have rigorously investigated the non-linear relationship between age at diagnosis and survival outcome. This study provides a key methodological contribution by demonstrating that the relationship between age and survival is complex and non-linear. We systematically tested and compared multiple statistical approaches, including linear, log-transformed, and restricted cubic spline models, to capture this relationship accurately. Furthermore, we transparently addressed a borderline violation of the proportional hazards assumption by employing a stratified Cox model, thereby strengthening the robustness of our key finding: that patients over 70 have a 3.3 times higher risk of mortality compared to the youngest patients.

The study concluded from its findings that younger MM patients had better survival rates, while older patients experienced worse outcomes. Age at diagnosis significantly affects survival, but its effect is not linear. A simple log transformation of age showed that survival worsens with increasing age, but did not fully capture the relationship. To better understand this, the researchers used a more advanced method (restricted cubic splines, in R), which confirmed a complex, nonlinear relationship between age and survival. The model confirmed that survival decreases as age increases, but not in a straight-line pattern. Statistical tests confirmed the model’s effectiveness. To investigate the proportional hazards assumption, we grouped patients by age and used a stratified Cox model. This improved the model and showed that older age groups had significantly higher risks of death, up to 3.3 times more for those over 70, compared to the youngest group.

One key limitation of this study is that it draws from a single-institution dataset (MMDB). While this offers a well-characterized and detailed patient cohort, it may restrict the generalizability of the findings to more diverse or broader populations. To address this, future research will focus on validating these results in independent, multi-institutional cohorts or large-scale national databases such as the SEER (Surveillance, Epidemiology, and End Results) registry. Furthermore, this study did not investigate potential effect modification by variables such as gender or race. Analyzing these interactions in future research is essential to understanding the nuances of the age–survival relationship across different patient subpopulations. Moreover, the accurately modeled non-linear effect of age established in this study can now be integrated with other critical prognostic factors such as cytogenetic risk profiles, ISS stage, and treatment data, to build a more robust and clinically actionable multivariable model.

Future research is needed to better understand the contributors to this observation, which could include disease-specific factors such as the relationship between disease characteristics and age of onset, factors not directly related to the disease such as the influence of comorbidities which are more prevalent with increasing age, and factors arising from both the disease and age such as stem cell transplant treatments which both affect the course of the disease and may be administered or not depending on age. Future research is also recommended that would include treatment types in survival analysis, as well as exploring targeted interventions that could enhance survival outcomes for older patients.

## Figures and Tables

**Table 1:** Hazard Ratios from Spline-Based Cox Model for Age at Diagnosis.

Spline Term	Coefficient	HR (Exp(Coef))	95% CI (Lower, Upper)	*p*-Value
Spline 1	1.8901	6.62	(2.19, 20.02)	0.0008
Spline 2	4.9336	138.88	(2.61, 7392.3)	0.015
Spline 3	2.6145	13.66	(5.79, 32.22)	2.36 × 10^−9^

**Table 2:** PH Assumption Results.

Test	Chi-Square	df	*p*-Value
ns (log of Age at Diagnosis, df = 3)	7.87	3	0.049
GLOBAL	7.87	3	0.049

**Table 3:** Stratified Cox Model Results.

Age Group	Coefficient	Exp (Coefficient)	Standard Error	*z*-Value	*p*-Value
40–49	0.1560	1.1689	0.1752	0.891	0.373049
50–59	0.5529	1.7382	0.1648	3.354	0.000797
60–69	0.7807	2.1829	0.1635	4.775	1.79 × 10^−6^
>70	1.1961	3.3070	0.1702	7.029	2.08 × 10^−12^

Number of patient observations: 2743. Number of events: 1619. Concordance = 0.577 (se = 0.007). Likelihood ratio test = 152.7 on 4 df, *p* < 2 × 10^−16^. Wald test = 150.1 on 4 df, *p* < 2 × 10^−16^. Score (log-rank) test = 156.8 on 4 df, *p* < 2 × 10^−16^.

## Data Availability

The approved IRB protocol for this study requires the raw data, which includes records of individual patients, though redacted to minimize personally identifiable information (PII), to be treated confidentially. Questions and requests related to the data may be directed to the authors.
